# Attitudes of Chinese College Students Toward Aging and Living Independently in the Context of China’s Modernization: A Qualitative Study

**DOI:** 10.3389/fpsyg.2021.609736

**Published:** 2021-05-31

**Authors:** Yan Zhang, Junxiu Wang, Yanfei Zu, Qian Hu

**Affiliations:** ^1^Institute of Sociology, Chinese Academy of Social Sciences, Beijing, China; ^2^School of Psychology, Inner Mongolia Normal University, Inner Mongolia, China; ^3^Shanghai University of Medicine and Health Sciences, Shanghai, China; ^4^Shanghai Cao Yang No. 2 High School, Shanghai, China

**Keywords:** aging, filial piety, individualization, modernization, living arrangement

## Abstract

Modernization in China is accompanied by some specific features: aging, individualization, the emergence of the nuclear family, and changing filial piety. While young Chinese people are still the main caregivers for older adults, understanding the attitudes of young Chinese people toward aging and living independently in the context of modernization is important because it relates to future elderly care problems in China. By using in-depth interviews and qualitative methods, 45 participants were enrolled in the study, 38 (84.44%) were women and 37 (82.22%) had no siblings. The ages ranged from 17 to 25 years (mean age = 19.28, SD = 1.74). Results revealed that participants held diverse attitudes about older adults, but the general attitudes were that older adults are lonely, financially disadvantaged, have poor social support, lack hobbies, and care about their children more than themselves. Chinese college students were affected both by traditional filial piety and individualism; however, of the two, they seemed put greater value on independence. Moreover, traditional filial piety is changing in a modern direction, affected by Western ideas of individualism: the status of the senior is diminishing, and living with one’s parents is no longer regarded as a necessary component. Implications concerning age stereotypes, elderly care policies, and strategies are discussed.

## Introduction

Modernization is a broad concept that refers to major social changes that occur when a preindustrial society develops economically, such as industrialization, urbanization, and bureaucratization (Zhang and Thomas, 1994). Since the reform and opening up in 1978, China has been moving toward modernization with some specific features. First, China is becoming an aging society (Bai, 2016). From 1999 to 2018, the number of senior citizens of 65 years or older in China increased from 86 to 166 million (from 6.9 to 11.9% of the total population; Logan and Bian, 1999; National Bureau of Statistics of China, 2019). However, low fertility since the late 1970s, especially in urban areas, led many young Chinese to not have siblings to share the traditional elderly care obligations, increasing their burdens (Bai, 2016).

Second, Western technology, political systems, and culture became a referential frame for the modernization of China (Rošker, 2014). Although some scholars attempted to preserve Chinese traditions regardless of modernization (Rošker, 2008), others considered modernization to be a transformation of essence in the sense of general social consciousness, production, and lifestyles (Li, 1986). For example, two research studies conducted in urban (Yang, 2015) and rural areas (Yan, 2011) in China have both claimed that the Chinese society is on the path toward individualization with the pursuit of privacy, independence, choice, and personal happiness being popularized and becoming the new family ideal for Chinese people. However, some research has held a middle ground. For example, Ji (2015) claimed that Chinese individuals are embracing modernization, individual identity, and independence and compromising tradition when necessary, although they still subscribe to patriarchal norms.

Third, modernization changed the power relationships in Chinese society (Zhang and Thomas, 1994). Modernization theory said modernization diminishes the status of older adults and disadvantages older generations (Cowgill, 1974). Consequently, people living in more modernized societies may hold more negative attitudes toward the aging people and the elderly than those living in less technologically developed countries (Bai et al., 2016). In traditional Chinese culture, people respect seniors, regarding them as having wisdom and authority (Hsu, 1953). However, an old Chinese saying: “suffering will occur if you do not listen to seniors’ advice,” has been restated nowadays by young Chinese adults as “happiness will last for years if you do not listen to seniors’ advice.” The Stereotype Content Model categorizes two dimensions of stereotype: warmth and competence (Fiske et al., 2002); and their cross-cultural study found that, globally, people regarded seniors as high in warmth but low in competence, including people from Hong Kong, China (Cuddy et al., 2008, 2009). Some studies have also shown that, although China is still on the path to modernization, the image and status of older people have already been negatively affected (Chow and Bai, 2011; Bai, 2016). Luo et al. (2013) found that Chinese students were more negative about their older adults than were their American counterparts, even though China is less modernized than the United States. This result cannot be explained by modernization theory. Vauclair et al. (2017) suggested that cultural differences in ageism are more nuanced than suggested by modernization theory. Other studies turned to the role of antecedents of ageism, such as knowledge and anxiety about aging (Donizzetti, 2019). Luo et al. (2013) also claimed that a lack of gerontological curriculum in the Chinese educational system, the caregiving burden faced by the one-child generation compounded with a lack of governmental support for caregiving, as well as the rising youth-oriented consumerist culture may account for Chinese students’ more negative attitudes toward the aging people and the elderly.

Fourth, modernization leads to the breakdown of the traditional extended family and the emergence of the “individualistic nuclear” family (Cowgill, 1986; Aboderin, 2004; Khalaila and Litwin, 2012). Yan (2009) said that the individualization of China leads to the pursuit of independence of youth, replacing the traditional “big family” ideal. Traditionally, children lived under the same roof with and took care of their aging parents (Hsu, 1948). Nowadays, the “empty-nest” living arrangement is becoming increasingly common for Chinese seniors, and the number of empty-nest elderly people who are living without children is increasing (Zhan et al., 2006). Some researchers found that compared to non-empty-nest elders (elders living with children), empty-nest elderly people had lower psychological well-being (Silverstein et al., 2006) and poorer mental health (Liu et al., 2007), more loneliness (Cheng et al., 2015), and depression (Zhai et al., 2015). However, other researchers found that empty-nesters were no different from non-empty-nesters concerning loneliness (Lin et al., 2009) and subjective well-being (Zhang, 2020); empty-nest elderly people were higher in subjective well-being (Liu et al., 2014) and life satisfaction (Sun, 2010; Poulin et al., 2014) than non-empty-nest elders. Although these research studies have produced inconsistent conclusions, some researchers blamed young Chinese adults for the emergence of empty-nest elderly people, accusing them of abandoning their filial piety obligations (Pu, 2014).

Filial piety (*xiao*) is an important cultural concept in traditional China and other Eastern countries. It requires children to make sacrifices for their parents to ensure the continuation of their parents’ happiness—not only by respecting older generations but also by taking care of aging parents by living together (Laidlaw et al., 2010). It was said that people in China were looking forward to entering into old age, where they will enjoy prestigious roles and statuses both within the family and in society (Bai, 2016). However, modernization theory maintains that, as society becomes modernized, filial piety diminishes (Cowgill, 1986). According to this theory, ageism may increase with modernization, and children may live independently with parents—leaving many elders as empty nesters. Yet, some researchers have not supported modernization theory completely. According to the Traditional-Modern Theory of Attitude Change (Dawson et al., 1971, 1972), when traditional cultural ideas conflict with modern culture, important traditional constructs will continue in the traditional direction, while unimportant traditional constructs will change in a modern direction. Filial piety is an important traditional construct in China, so it should continue in a traditional way. However, research has found that, explicitly, this was the case but, implicitly, filial piety was changing in a modern direction, affected by modern individualistic western-influenced cultural ideas (Zhang et al., 2021). Bedford and Yeh (2021) discussed the evolution of the conceptualization of filial piety and developed the Dual Filial Piety Model. This model suggests two kinds of filial piety: reciprocal and authoritarian (Yeh, 1997; Bedford and Yeh, 2021). Reciprocal filial piety is defined as affection-based gratitude and respect for parents’ efforts, while authoritarian filial piety refers to the relationship hierarchies and role obligations that demand children’s compliance with their parents (Yeh and Bedford, 2003; Bedford and Yeh, 2021). Yeh and Bedford (2003) found that college students had higher levels of reciprocal filial piety than authoritarian filial piety. Feng (2013) argued that Chinese filial piety is going to be more reciprocal and less authoritarian; young Chinese adults will still respect elders but will not completely obey them.

Aforementioned contradictory results were almost all from quantitative studies using predetermined, closed-ended questions about the attitudes of the participants. These methods may limit the participants’ answers and fail to shed light on their attitudes. In the context of Chinese modernization, aging and low fertility increased elderly care burdens on young Chinese people. Young Chinese people are still the main force of elderly care and will continue this in the near future because, to date, there is no functioning non-familial elderly care system in China (Tu, 2016) and 90% of seniors still rely on familial care (Zhang, 2012). Will young Chinese people be ageist? Will they hold onto some positive attitudes about seniors and seniors’ attitude dimensions apart from warmth and competence? If so, what would these attitudes be? Moreover, what exactly are the attitudes of young Chinese adults toward living independently from their parents? Are they less willing to take care of seniors and live together? In general, will modernization lead young Chinese people to be less filial? Understanding these questions is important because they relate to future elderly care problems in China. However, we need more exploratory methods to answer these questions. We assumed that, in the context of modernization and between the conflicts of traditional and modern cultures, answers may be more complicated than filial versus unfilial, ageist versus not ageist, prefer to live independently versus do not prefer. Qualitative methods offer us a way to hear about the different attitudes and the various voices among young Chinese people through open-ended questions about both common attitudes and rare attitudes.

## Materials and Methods

### Participants

Participants were college students from a university in Shanghai, China. We briefly introduced the purpose and method of the study before several classes in the university. Students who were willing to enroll could either write down their contact information to researchers immediately after the introduction or send a message to the researchers at any other time. In total, 45 participants voluntarily engaged in the study: 38 (84.44%) were women and 37 (82.22%) had no siblings. The ages ranged from 17 to 25 years (mean age = 19.28, SD = 1.74 years). More characteristics are shown in Table 1 and Supplementary Table 1.

**TABLE 1:** Participants’ characteristics.

Information gathered by survey			Information extracted from interviews		
	*N*	%		*N*	%
**Age**	19.28 ± 1.74		**Only one child**		
**Gender**			Yes	37	82.22
Male	7	15.56	No	8	17.78
Female	38	84.44	**Motivation for attending the study**		
**Year in college**			Offer help	4	8.89
1st	28	62.22	Money reward	3	6.67
2nd	2	4.44	Relationship with researcher	4	8.89
3rd	13	28.89	Interested in elders	22	48.89
4th	2	4.44	Interested in research methods	12	26.67
**Hometown**			**Grandparents’ living place (father’s side)**		
Urban areas	27	60.00	Urban	20	44.44
Rural areas	18	40.00	Rural	25	55.56
**Raised mainly by whom**			**Grandparents’ living place (mother’s side)**		
Parents	26	57.78	Urban	21	46.67
Grandparents	14	31.11	Rural	24	53.33
Both	5	11.11	**Grandparents’ living arrangements (father’s side)**		
**Contact frequency with elders**			Living with children	12	26.67
A little	10	22.22	Living without children	28	62.22
Some	26	57.78	Died/not clear	5	11.11
Frequent	9	20.00	**Grandparents’ living arrangements (mother’s side)**		
**Familiarity with empty-nest elders**			Living with children	16	35.56
No idea	3	6.67	Living without children	22	48.89
A little	17	37.78	Died/not clear	7	15.56
Some	16	35.56			
Very much	9	20.00			

### Researchers

YZh (a woman, 33 years old), JT (a woman, 23 years old), and TL (a man, 23 years old) served as the primary research team; and YZu (a woman, 41 years old) and QH (a woman, 29 years old) provided an outside audit to check on the findings of the research team. In qualitative research, it is crucial that researchers address possible biases that might contaminate the coding and analysis of the data (Stiles et al., 1990). Although they may be firmly committed to honoring the data, no researchers are without bias. Therefore, we tried to articulate these biases at the outset of the study by exploring and discussing our attitudes toward aging and living with parents, as well as our research hypotheses in the team, setting them aside during the analyses, and reflecting on their effect on the analyses by writing reflection memos and discussing these with team members, as has typically been suggested for those involved in qualitative research (Hill et al., 1997).

For readers to evaluate the validity of results, we share the attitudes of the research team: YZh said she agreed that living independently with children is not necessarily bad for old parents, but it could be an active choice of old parents; JT said she thinks being old is unimaginable and lonely, she wants to be with family when she is old, so she also wants to accompany her parents when they are old; TL said he wants to be a filial son and wants to have his own career, but he thinks the two are not contradictory, because living a good life is another kind of filiality. YZu said she believes that young Chinese should respect seniors, but it was unnecessary that they should live together because there will be lots of familial conflicts by living together; and QH said she thinks people could age gracefully and be productive and old parents should not live together with children, when old parents could not take care of themselves, children could hire senior workers to take care of their parents.

### In-depth Interviews

A demographic form about age, hometown, etc., was provided to interviewees to fulfill before interviews. Semi-structured interview protocols were used with all interviews. Modifications were made after several interviews. Questions in the final protocol are: (1) Motivations to attend this study, e.g., “Why do you want to attend this study?” (2) Attitudes toward older adults and aging, e.g., “What are your perceptions of older adults and aging?” (3) Attitudes toward living independently, e.g., “What are your perceptions of older adults living without children?,” and (4) Coping with aging in the future, e.g., “What will you do when your parents/you become old in the future?”

Each member of the primary research team interviewed 15 participants in Chinese. Each interview lasted from 20 to 50 min. After an interview was finished, it was transcribed (excluding minimal responses, e.g., “Hmm”; but including special situations, e.g., long pause) and analyzed in a week by the interviewer. Transcripts were assigned a code number to maintain confidentiality.

### Data Analysis

Data were managed and analyzed according to Consensus Qualitative Research Methods (CQR; Hill et al., 2005) in NVivo 12.0 (QSR, Australia).

#### Case Summary

Before coding a certain transcript, a research member read the transcript briefly and created a case summary, which included about 200 words describing the attitudes of aging and living independently of the participant, as well as the reader’s initial ideas and feelings after reading the transcript. Although Hill et al. (1997) recommended keeping memos or notes about impressions and comments immediately after the interview, we wrote case summaries after transcription (Miles and Huberman, 1994). The reason for this was that emotional involvement after an interview could sometimes be overwhelming. Case summarizing after transcription provided “a step” distance, creating an emotional connection without getting too involved. However, this does not mean that researchers were forbidden from keeping memos after the interviews. If they wanted to, they were free to do so. These memos and case summaries worked as both search tools for us to search basic information of participants quickly and reflection memos because initial thoughts and feelings were more intuitive, less susceptible to bias and research expectations, and more conducive to finding situations that are different from what we assumed.

#### Coding of Themes

The primary team members examined the first three transcripts individually and placed each block of data related to the same idea into initial themes. Later transcripts were coded according to these initial themes, but when initial themes were not applicable, either theme was modified or a new theme was generated. Disagreements about themes and how to block the data were discussed until the team reached a consensus, which was audited by YZu and QH and then reviewed again on the basis of their comments. Four major themes were identified: (1) diverse attitudes toward older adults, (2) perceptions of reasons that lead to unhappiness/happiness in old age, (3) attitudes toward living with and without children, and (4) attitudes and coping strategies when parents/themselves are old. Results were translated into English.

#### Coding of Categories

After blocking data into themes, the three primary team members independently read data within a given theme and coded them sentence by sentence. Each member then categorized codes and devised hierarchical categories. After that, the team met as a group to discuss their codebooks and to arrive at a consensus about the categories and how to word them. The themes and categories were continually modified throughout the process to reflect the team’s ongoing understanding of the data.

#### Audit

Codebooks were then sent to the two auditors (YZu and QH) who read them, suggested additions and deletions and returned them to the primary team for further discussion and revision. The primary team then reviewed each case to make sure they had been consistent over time and to be certain that they had remained true to the participants’ perspectives. Revisions were made to the themes and about what was included in the themes for each case. The team added notes where appropriate to help them remember issues and questions that occurred to them, but these were not directly stated in the data. Then every case was examined again by the team members, who arrived at a consensus over changes.

#### Cross-Analysis

Using the consensus versions of the codebooks, the primary team met and conducted cross-analysis for all transcripts. The purpose of doing the cross-analyses was to compare the codebooks to determine consistency across the transcripts. We also wanted to ensure that we were applying the same criteria across transcripts.

We reviewed each theme separately and evaluated categories. When a category was identified for certain cases, we returned to the raw data to determine whether the same category also fits other cases. During this process, the team developed dimensions for some categories. The team then calculated the number of participants who mentioned a certain dimension of each category for comparison, although some researchers did not support counting exact numbers because participants were not asked the same questions in the same way; therefore, counting numbers could be misleading (Sandelowski, 2001; Neale et al., 2014). Nonetheless, we counted the number of participants rather than using the labels “general” (if a category occurred in all cases), “typical” (if a category occurred in at least half of the cases), and “variant” (if a category occurred in only one or few cases) as in CQR to replace numbers. This was the case because the number of participants in our study was much larger than in the regular CQR process so that most of the categories were “typical.” Thus, only showing labels could not provide enough information for comparison. However, using numbers was not meant to convey generalizability beyond the study population.

#### Stability Check

After we interviewed the 40th participant and analyzed the transcripts, no new themes or categories were added, assuming that the data were saturated or stable. Even though Hill et al. (2005) recommended a sample size between 8 and 15 participants (Hill et al., 2005), we interviewed more participants, because we could not reach saturation with 15 participants. Moreover, to check whether the data were truly saturated, five more interviews were conducted. Each of the categories for the five cases was placed into the existing cross-analysis by the two auditors. No new categories were added. The new cases only changed the frequency of each category. Consequently, we determined that the data were stable and that additional cases were unlikely to change the results in any significant way. In total, we conducted 45 interviews.

#### Bias Control

To ensure greater credibility, first, each team member was conscious of her or his attitudes about aging and living independently. Second, each researcher wrote independent methodological and reflective memos about the impact of her/his attitudes on the collected data. Third, the team met on a weekly basis to discuss the degree to which the researchers attitudes might have influenced the analysis of the data and the level of openness of the participants. Fourth, the team discussed data analyses and translations weekly and reached a consensus on both the results and the translations (Rhodes et al., 1994; Hill et al., 1997, 2005).

### Compliance With Ethical Standards

According to the accepted research standards incorporated by this study, the privacy of each participant interviewed has been protected. The research team did not use the real names of the participants, nor any identifiable information. All participants were aware that they were participating in a study, knew the research objectives, and consented to participate in the qualitative research. It was explained to the participants that their participation was totally voluntary and if they chose not to participate or answer certain questions there would not be any negative repercussions. Signed informed consent was obtained for both the interview and audiotaping. Two participants did not consent to be audio-taped; however, their signed consent for notetaking was obtained. At the conclusion of the interviews, the participants were thanked for their participation and contribution to the research and were offered 15 RMB (about two dollars) for participating.

## Results

Results were translated into English; the team reached a consensus on both the results and translations. Quotations are followed by the identifier of the participant. For a clearer presentation of results, Supplementary Tables 2–5 are provided in Supplementary Materials. In these Tables, comparisons are shown along the same rows. For example, in Supplementary Table 2, the two opposite phrases “lonely” and “not lonely” are in the same row. The number of participants that mentioned “lonely” was 15, whereas only one participant mentioned “not lonely.” Thus, the readers can compare the two opposite attitudes about older adults easily and see that more participants said that older adults are lonely.

### Diverse Attitudes About Older Adults

We divided participants’ attitudes about older adults into four categories: attitudes concerning physical/mental health, quality of life (QoL), social support, and personality of older adults. We also assigned valence to each attitude as negative, positive, neutral, or unclassifiable based on the information provided by participants and research members reached a consensus on the valence assignment.

#### Attitudes About Older Adults’ Physical/Mental Health

More participants held a negative attitude about the physical and mental health of seniors than those who held a positive attitude. Some participants mentioned that they think seniors are unhealthy physically (*n* = 5), lonely (*n* = 15), unhappy (*n* = 3), anxious (*n* = 2), feeling hopeless (*n* = 1), and useless to their families and society (*n* = 3); in contrast, only few participants said seniors are healthy physically (*n* = 2), happy (*n* = 5), and not lonely (*n* = 1).

#### Attitudes About Older Adults’ Quality of Life

We categorized the quality of life (QoL) of seniors into four subcategories: overall QoL, busy with labor work, richness of leisure activities, and economic affluence.

##### Overall QoL

One participant said elders’ overall QoL is just so-so, not too bad, not too good; some participants said this kind of life suits seniors well (*n* = 7). *“Even though their lives are not that colorful, with less vigor and vitality, compared to our young people, it suits them well.” (#6)* However, the sentence may imply participant’s latent attitudes that colorful lives did not suit seniors.

##### Busy with labor work

Participants’ attitudes lay on a continuum from idle (having nothing to do) to at ease (having something but not too many things to do) to laborious (having too many things to do). These attitudes were held by nearly the same number of participants (idle: *n* = 11, at ease: *n* = 10, laborious: *n* = 10). In this continuum, idle and laborious were problematic in the eyes of the participants, while at ease represented a good living status. However, six participants considered that seniors want to keep themselves busy, although they did not need to do things. From these participants’ views, laborious work is not a bad thing because it is seniors who chose to do things rather than being forced to do them.

##### Richness of leisure activities

Several participants mentioned that seniors have rich leisure activities (*n* = 5); however, many participants described older individuals’ lives as in a low or medium level of richness but used different words. As for the low level of richness, some participants described it by using words like “boring” (*n* = 16), which reflected a negative attitude in Chinese; while some used words like “simple” (*n* = 11), which reflected a positive attitude in Chinese. As for a medium level of richness, some participants described it by using the word “repetitive” (*n* = 2), which reflected a negative attitude in Chinese; while some participants described it by using words like “regular” (*n* = 12), which reflected a neutral or positive attitude in Chinese.

##### Economic affluence

Several participants mentioned the economic affluence level of seniors; however, they used words in opposition: affluent (*n* = 2) and poor (*n* = 4).

#### Attitudes About Social Support for Older Adults

All the five participants who mentioned this category held negative attitudes concerning the social support for older adults. They said seniors have bad marital relationships (*n* = 2), have nobody to talk to (*n* = 1), have few friends (*n* = 1), and have no constant companions, for example, children (*n* = 1).

#### Attitudes About Personal Traits of Older Adults

Participants used several contradictory words/phrases to describe seniors: closed (*n* = 7) and open (*n* = 1), have no hobbies (*n* = 6) and have many hobbies (*n* = 3), lazy (*n* = 3) and industrious (*n* = 6), self-centered (*n* = 3) and do not care about the self but only care about children and family (*n* = 14), dirty (*n* = 1) and clean (*n* = 1), and dependent (*n* = 4) and independent (*n* = 3). Further, seven participants mentioned thrift, one mentioned brave, and another mentioned nagging.

In sum, participants expressed diverse attitudes about older adults, and some participants’ attitudes were in opposition or they used varied terms to describe the same thing. However, according to the categories mentioned most by the participants, we summarized participants’ general attitude toward the seniors as follows: lonely, live lives with a low or medium level of richness, have poor social support, closed, have no hobbies, industrious, thrifty, do not care about themselves but only care about children and family.

### Perceptions of Reasons for Unhappy/Happy Late Life

Since participants held diverse yet general negative attitudes toward seniors, what do they think are the reasons why seniors were unhappy and what makes seniors happy? Five categories emerged out of participants’ perceptions of reasons for unhappy/happy late life. Four categories were the same as above: physical/mental health, QoL, social support, and personal factors. The novel category was society.

#### Physical/Mental Health as a Reason for an Unhappy/Happy Late Life

In this category, physical health ranked first as the main reason for happiness (*n* = 11) and unhappiness in old age (*n* = 6). Moreover, for some participants, feeling lonely (*n* = 1) and losing control (*n* = 1) were reasons for unhappiness, while feelings of belongingness (*n* = 3) and being respected (*n* = 2) were reasons for happiness.

#### Quality of Life as a Reason for an Unhappy/Happy Late Life

Economy was the chief reason mentioned by several participants as a reason for unhappiness (*n* = 6) and happiness in old age (*n* = 5). Other participants said that having hard work to do (*n* = 3) or having nothing to do (*n* = 1) were both harmful to older adults’ happiness. Not needing to work (*n* = 5) but having a rich life (*n* = 4) were reasons for perceived happiness, which, again, indicates the difference between a *choice* and being *forced* to do something. Other descriptions of happiness in old age included living a regular (*n* = 2), quiet and peaceful life (*n* = 1) without having big stressful life events (*n* = 4).

#### Social Support as a Reason for an Unhappy/Happy Late Life

Over half the participants mentioned bad social support as the reason for unhappiness in old age (*n* = 31). In this category, children’s lack of filiality was the chief reason (*n* = 25), including not being around, not accompanying or visiting aging parents, having bad relationships with one’s parents, or bringing shame to the family. Some participants did not separate children from other family members and they used the term “family” generally. Having family conflicts may lead to unhappiness in old age (*n* = 8). Other reasons mentioned by participants that made older adults unhappy were living alone (*n* = 4), the death of a partner (*n* = 1), and living in nursing home (*n* = 1).

In contrast, 27 participants mentioned good social support as the reason for happiness in old age (*n* = 27). Children’s filiality was the chief reason (*n* = 21), includes being around, accompanying or visiting aging parents, having good relationships with one’s parents, or bringing glory to the family. Having good family relationships (*n* = 7), having friends (*n* = 7), having good neighborhood relationships (*n* = 3), and having people to accompany them (*n* = 1) may also make older adults happy.

#### Personal Traits as a Reason for an Unhappy/Happy Late Life

Notably, more participants mentioned personal traits of seniors as reasons that made older adults happy (*n* = 27) than those who mentioned personal traits as reasons that made older adults unhappy (*n* = 10). “Having hobbies” ranked first in personal factors for happiness in old age (*n* = 15), while only three participants mentioned “having no hobbies” as a reason for unhappiness in old age. Other personal traits associated with unhappiness and happiness included being closed (*n* = 3) and keeping pace with the time (*n* = 2), respectively; not content (*n* = 1) and content (*n* = 5), respectively; pessimistic (*n* = 1) and optimistic (*n* = 5), respectively; and bad character (*n* = 3) and good character (*n* = 1), respectively. Other personal factors for happiness in old age were having an ability (*n* = 5; e.g., high education), having faith (*n* = 1), and being resilient (*n* = 1).

#### Society as a Reason for an Unhappy/Happy Late Life

This category was only mentioned by one participant as a reason for unhappiness in old age. *The reasons for an unhappy late life is because—not only the children—however, also the outside society. The whole society treats the elders unfairly; they did not enjoy the benefits they should have enjoyed* (#25).

Notably, four participants said that they did not notice any older adults being unhappy; they just saw unhappy older adults in the news. However, one participant was negative about aging and said that all older adults were the same—no one is happier, and no one is unhappier.

In sum, participants’ reasons for happiness and unhappiness in old age were almost the same in both content and frequency. However, participants considered personal factors (especially having hobbies) to be more important for happiness in old age as compared to unhappiness.

### Attitudes Toward Living With and Without Children

Since traditional filial piety is deep-rooted in Chinese culture, and participants ranked children’s filiality as the chief reason for the happiness of seniors, we then analyzed participants’ attitudes toward seniors living with or without children. Participants compared the cons and pros of the two kinds of living arrangements and considered that they were different in the four categories: mental health, QoL, social support, and personality.

#### Attitudes Toward Influences of Living Arrangements on Older Adults’ Mental Health

Many participants stated that it is better for old parents’ mental health to live together with children than to live separately from children. First, some participants (*n* = 12) mentioned that elders living with children were happier than elders living without children because they have children to depend on (*n* = 10), were less lonely (*n* = 6), do not have a sense of loss (*n* = 2), do not need to suffer from missing their children (*n* = 1), and do not need to worry about their children (*n* = 1). Some participants said that living with children helps elders feel spiritually supported by children (*n* = 10), which may also help elders to feel safe (*n* = 1). Two participants said that, when living with children, elders need to help children do some household duties. This may bring elders a feeling of being useful. One participant said, “*Living with children brings older adults a feeling of superiority because her children could take care of her*” (#1). Another participant said, “*Living without children may lead to some mental illness*” (#3). On the contrary, only one participant stated that it is better for elders’ happiness to live separately from their children because “*they have more freedom; they do not need to depend on children; [and] they can decide what to eat, to buy, when to eat, [and when] to sleep*” (#41).

Two participants said that whether an older adult is happy depends on whether his/her children are filial rather than whether his/her children are living with them. Moreover, eight participants stated that being old is lonely, regardless of who you live with, because children are too busy to care for elders regularly: “*Although she* (*grandmother of father’s side*) *lives with us, my parents have to work, and I have classes. We only meet in the evenings. [In] a whole day, what can she do at home? She could not read; she could not watch TV. The only thing she could do is sit there*” (#1).

#### Attitudes Toward Influences of Living Arrangements on the QoL of Older Adults

Concerning QoL, three participants said life is more colorful if children are around, another three participants said living with children gives elders things to do, and one participant said living with children enriches elders’ lives. In contrast, three participants regarded living with children as a disturbance to elders, and another three participants said it burdened elders. They said elders should be taken care of by their children; however, when living with children, some elders had to take care of their children instead (e.g., cooking): “*The nanny with grandchildren…although she lives with children and her husband, she does all household duties. I feel she’s tired, and she’s getting older and older; her children won’t help…I think she may have some resent…but, another nanny, because she didn’t live with her children, if she wants, she could also go to visit her children. Usually, she visits neighbors, [I] feel she’s very happy*” (#28).

#### Attitudes Toward Influences of Living Arrangements on Older Adults’ Social Support

Two participants said it is better for family relationships if elders are living with children; while one participant said that if they live together, there will be lots of mother and daughter-in-law conflicts, so it is worse for family relationships.

#### Attitudes Toward Influences of Living Arrangements on Older Adults’ Personality

One participant stated that living with children helps elders to be modern and keep pace with the time, although one participant said that it depended on elders and that older adults can have their own lives regardless of their living arrangement.

In sum, more participants thought that living with children was better for elders’ mental health than living without children; however, there were perceived equal pros and cons concerning QoL, social support, and personal factors.

### Attitudes and Coping Strategies When Parents/Themselves Are Old

Results before were general attitudes and perceptions of aging and living independently of participants, but in the last part, we invited participants to take a more “insiders”’ view, talking about their parents’ aging and their own aging, including living independently from their parents when their parents are old, and living independently from their children when they themselves are old. Below, we compare participants’ attitudes and coping strategies separately.

#### Attitudes Toward Living Independently From Parents When Parents Are Old

At the time of interviews, many participants were studying away from home; however, many could not accept separating from their parents when their parents were old (*n* = 23). They said they would not let their older parents live alone (*n* = 16) because they would worry about them (*n* = 1). Ten participants said they could accept living separately from their old parents because it is an evitable trend (*n* = 5), they did not want to live with them (*n* = 2), and parents have their own lives (*n* = 1). Two participants said that their parents could adapt to living without children nearby when they are old. Furthermore, three participants were struggling with this issue: on the one hand, they wanted to take care of their parents; on the other hand, they wanted to have their own lives and pursue their dreams.

#### Attitudes Toward Living Independently From Children When Participants Themselves Are Old

Many participants said they can accept living without children when they are old (*n* = 34) because it is an evitable trend for parents and children to live separately in the future (*n* = 4), they do not want to live with children (*n* = 9), or they do not need to live with children (*n* = 13). Nine participants said it is good for children to live separately because it reduces family conflicts and children can focus on individual development. Ten participants could accept living separately from children conditionally, as long as their children are living nearby (*n* = 5), children visit often (*n* = 4), children are filial (*n* = 1), and they have others to accompany them (*n* = 1). Moreover, three participants reluctantly accepted living separately from children: “*if they do have to go outside to work, I can’t force them to live with me…just live my own life*” (#16). Only one participant said she cannot be apart from her children when she is old because “*the parent–child relationship is the most important thing in the world. Blood is thicker than water*” (#42).

#### How to Take Care of Parents When Parents Are Old

Participants provided several strategies to care for aging parents. The strategy mentioned by the most participants (*n* = 31) was “try my best;” that is, participants would try their best to balance the needs of parents and themselves—the needs of filial piety and independence. This strategy included visiting parents often (*n* = 20), staying close to home (*n* = 6), living in the same city (*n* = 3), traveling with parents (*n* = 3), calling parents on the phone (*n* = 3) or video calls (*n* = 3), asking others to take care of parents (*n* = 3), and buying things for parents (*n* = 1). However, the “try my best” strategy implies that they would not live with their parents.

The second strategy was to change parents (*n* = 12), which includes encouraging parents to have their own lives (*n* = 9): “*[I] tell them to find some hobbies or go outside*” (#2) and move to the city where the participants might live (*n* = 3): “*Maybe I’ll pick them up if I’m living in a city someday. I’ll try not to let them live alone*” (#4).

The third strategy was the “if…then” strategy (*n* = 10); that is, participants will make choices about how to take care of parents according to varied situations. For example, “*if I have a good development, I will take parents over to the place I live; if I have a bad development, I will go back to the place my parents live*” (#43).

The fourth strategy was to change themselves (*n* = 3). They said they would give up their career in big cities to go back home and accompany their parents when they are old. One participant said that no matter what happens, she will not live with her parents when her parents became old (#25); and one participant said he will try to reach an agreement with his parents about their living arrangement (#12).

#### How to Be Happy When Participants Themselves Are Old

According to most participants (*n* = 29), when they are old, they will depend on themselves for happiness. Only two participants said that they would try to educate children to be filial to ensure happiness. Many participants said they would get hobbies (*n* = 22), such as traveling, handcrafts, music, and painting. Some participants (*n* = 10) said they would make friends expand their social circle beyond their children. Five participants said they would have a good socioeconomic status because that determines QoL.

In sum, although many participants mentioned that they cannot accept living separately from their parents when their parents are old, many could accept living separately from their children when they themselves are old. As for coping strategies, most participants did not actually consider living with parents in the future. They are struggling between filial piety and independence; however, it seems that independence wins. Participants prefer to depend on themselves rather than on their children for a happy later life.

## Discussion

First, participants held diverse attitudes about older adults. However, we did find that participants regarded seniors as low in competence and high in warmth as claimed by the Stereotype Content Model (Fiske et al., 2002). Instead, the four dimensions we divided in the results were physical/mental health, QoL, social support, and personality. The results suggested a need for an open-ended method in future studies about stereotypes or ageism. Moreover, results revealed that participants held diverse attitudes about older adults, but the general attitudes were that older adults are lonely, financially disadvantaged, have poor social support, lack hobbies, and care about their children more than themselves. It appears that our study supported that young Chinese people held negative attitudes toward older adults. However, was this because of modernization or other antecedents?

An antecedent of ageism is the anxiety of aging (Donizzetti, 2019). From a developmental perspective, being independent is an important task. Since college students have just gained independence, imagining returning to live with one’s parents or future children may be a source of anxiety, which then reinforces negative attitudes toward aging. However, whether being independent is an important developmental milestone is culturally dependent. Many researchers have shown that traditional Eastern countries emphasize interdependence more than independence (Hsu, 1953; Shweder et al., 1984; Markus and Kitayama, 1991). Jensen (2008) outlined the cultural-developmental template to illustrate that the development of three Ethics—Autonomy, Community, and Divinity—varies across cultures. She gave an example of religious conservatives and showed that there may be some decrease in the Ethics of Autonomy over their lifespan because of the emphasis on renouncing self-interest. Inference in reverse, the negative stereotype in our research may still be a result of modernization, which leads to the individualization of China and the increasing need for independence among young Chinese people.

Another antecedent of ageism is the knowledge of aging (Donizzetti, 2019). However, we will argue that it may not be the insufficient knowledge of aging but the “wrong” knowledge of aging that leads to ageism. In our results, participants regarded personality, such as being independent, modern, and keeping pace with the times as positive and considered personal traits, especially having hobbies to be more important for happiness in old age as compared to unhappiness. This is in accord with the dialogs of active aging that being old is not necessarily negative, while seniors could be active, healthy, independent, and productive (Carr and Weir, 2017). The new dialogs of aging are sweeping the world in modernized areas; however, it has an implication for individualism by applying a prescription that independence is normative and “good” in old age (Ranzijn, 2010). Ranzijn (2010) argued that the active aging dialogs may paradoxically reinforce negative stereotypes of seniors as ill, dependent, and non-productive; it is also underpinned politically and economically to empower individual responsibilities but reduce the burden on family and society.

Second, it seems the two contradictory values—filial piety and independence—coexist in Chinese young adults. For example, we found that children were the most important reason for older adults’ happiness or unhappiness. Living with children was considered better for elders’ mental health than living without children. These results implied that young Chinese adults may still be affected by filial piety—the responsibility to ensure old parents’ happiness. This was confirmed again in the results that participants could not accept living separately from their parents when their parents are old. On the contrary, many of them could accept living separately from their children when they themselves are old. Moreover, they prefer to depend on themselves rather than their children for a happy later life. This phenomenon has also been found in other cultures. For example, Mount (2017) found that middle-class Indian women were pressured to conform to the facets of traditional womanhood while also aligning themselves with modernity.

However, when tradition meets modern, what will win? The Traditional-Modern (T-M) Theory of Attitude Change argues that individuals are motivated to reduce the conflicts between traditional and modern cultures by changing their attitudes to semi-traditional or semi-modern: more “important” traditional concepts will change in a traditional direction, while the “un-important” concepts will change in a modern direction (Dawson et al., 1971, 1972). Since filial piety is ranked as the most important traditional concept by both lay people and experts in China (Zhang and Weng, 2017), according to T-M theory, it should change in a traditional direction; that is, people may place more value on filial piety when traditional meets modernity. However, our results refuted this speculation and revealed the superiority of independence, which was consistent with the findings of Zhang et al. (2021) that implicitly, all traditional concepts are changing in a modern direction, affected by Western individualistic ideas.

For example, some participants said regardless of who you live with, being old is lonely because children are too busy to take care of the old parents. Instead, seniors had to take care of their children if they were living together. It seems that the relationship between children and parents is changing in a direction as suggested by modernization theory that modernization diminishes the status of seniors (Cowgill, 1974, 1986). Moreover, although many participants mentioned that they cannot accept living separately from their parents when their parents are old, most participants did not actually consider living with their parents in the future. They said they will try their best to visit parents often, stay close to home, live in the same city, and make phone/video calls. However, the “try my best” strategy implies that parents will be sacrificed, while filial piety requires children to sacrifice. Nonetheless, we do not think this implies the death of filial piety, as some researchers have claimed (Yan, 2011). Although preferring not to live together, our participants said they would still be filial to parents in other ways. Therefore, perhaps just the construct of filial piety has changed. Living together with parents was not regarded as a necessary component of filial piety. One survey from the 1990s showed that Chinese residents preferred to live close to, but not necessarily with, one’s parents (Tu, 2016). Prior results of dual models of filial piety found the weakening of authoritarian filial piety but the maintenance of reciprocal filial piety (Yeh and Bedford, 2003; Feng, 2013).

In conclusion, our results suggest that Chinese college students are affected both by traditional filial piety and individualism; however, of the two, they seem place greater value on independence. Moreover, traditional filial piety is changing in a modern direction, affected by Western ideas of individualism: the status of older people is diminishing, and living with one’s parents is not regarded as a necessary component.

### Implications

The study had several implications. First, our results suggested that young Chinese people held generally negative attitudes toward aging in four dimensions: physical/mental health, QoL, social support, and personal factors rather than in the two dimensions of the Stereotype Content Model—warmth and competence (Cuddy et al., 2008). Further studies about stereotypes should not be limited in their scope and could use more open-ended methods. Second, we should be cautious about active aging dialogs by assuming that being healthy and independent are normative in old age and thus reinforcing negative stereotypes of seniors as ill and dependent (Ranzijn, 2010). Third, young Chinese adults prefer to be independent and live independently from their parents; therefore, we predict that the Chinese elderly care model will become more Westernized in the future. However, non-familial-care in China is underdeveloped, and the increasing number of empty-nest elderly people will require care (Tu, 2016). Consequently, the Chinese government and non-government institutions should determine how to develop non-familial care for elders. Fourth, young Chinese adults are also affected by traditional filial piety, but the content of filial piety is changing to compromise with the need of pursuing personal development and happiness. Thus, we could accommodate the change by providing children more opportunities to live near their parents instead of living with their parents, to video chat with them, and to pay visits to their parents.

### Limitations and Strength

This study had some limitations. Its main limitation is sample bias. As a qualitative study, it only enrolled a small number of participants. Specifically, participants came from a university in Shanghai, were in their 20s and many of their parents were in their 40s, and most of them were female and an only child. Therefore, this study’s generalizability is limited and its results cannot be generalized to other groups. This limitation may be inevitable for all qualitative research. Here, qualitative research findings are not intended to be generalized, but rather they aim to shed light on the attitudes or experiences of participants, revealing other voices. Our research also aims to share the various attitudes toward aging and living with parents of Chinese youth; however, whether these voices are representative of all Chinese youth is still questionable. The risk of sample bias means that we should be cautious about our conclusions, which could be reexamined by quantitative studies in the future. Second, we presented some information that we think may affect the validity of the results, such as participants’ motivation for taking part in the study and researchers’ gender, age, expectations, and bias; however, how they may affect this study’s validity is unclear. Future qualitative methodology studies could explore these aspects more deeply. Yet, providing background information is still important for readers to grasp the context of the “story.” Third, participants answered questions about their assumed attitudes and coping strategies when their parents and themselves get old; longitudinal studies that address participants’ attitude changes in the future should be considered.

Despite these limitations, this study advanced an exploration of the attitudes of young Chinese adults about aging and living independently and, more specifically and deeply, helped to better understand Chinese youth undergoing social change. The study also suggested other dimensions of age stereotyping and methods to study stereotypes, pointing out the potentially negative effect of active aging on ageism and future elderly care models and strategies.

## Data Availability Statement

The datasets presented in this article are not readily available because some interviews may include identifiable personal information. Requests to access the datasets should be directed to YZ, yanzhangpsy@126.com.

## Ethics Statement

The studies involving human participants were reviewed and approved by East China Normal University. The patients/participants provided their written informed consent to participate in this study.

## Author Contributions

YZh contributed on research design, interviews, data analysis, and manuscript writing. JW contributed on research design, theory construction, and manuscript revising. YZu contributed on participants recruitments, interviews, and data analysis. QH contributed on interviews and data analysis. All authors contributed to the article and approved the submitted version.

## Conflict of Interest

The authors declare that the research was conducted in the absence of any commercial or financial relationships that could be construed as a potential conflict of interest.
