# Supplementary material for: Attitudes of Chinese College Students Toward Aging and Living Independently in the Context of China’s Modernization: A Qualitative Study
Source: Front Psychol. 2021 May 31;12:609736. doi: 10.3389/fpsyg.2021.609736 (PMC8200472; doi:10.3389/fpsyg.2021.609736)
Supplement: Supplementary file 2 [file Table_2.DOCX]

**Supplementary Table 2. Comparison of attitudes to older adults (*N* = 45).**

|  |  | **Negative attitudes** | ***N*** | **Positive attitudes** | **N** | **Neutral/Unclassifiable** | ***N*** |
| --- | --- | --- | --- | --- | --- | --- | --- |
| **Physical/mental health** |  | lonely | 15 | not lonely | 1 |  |  |
|  |  | unhealthy in body | 5 | healthy physically | 2 |  |  |
|  |  | unhappy | 3 | happy | 5 |  |  |
|  |  | anxious | 2 |  |  |  |  |
|  |  | feeling hopeless | 1 |  |  |  |  |
|  |  | feeling useless to families and society | 3 |  |  |  |  |
| **QoL** | overall |  |  |  |  | so-so | 1 |
|  |  |  |  |  |  | suitable to the old | 7 |
|  | busyness | idle | 11 | at ease | 10 |  |  |
|  |  | laborious | 10 |  |  | want to work | 6 |
|  | richness | boring | 16 | simple | 11 |  |  |
|  |  | repetitive | 2 | regular | 12 |  |  |
|  |  |  |  | rich | 5 |  |  |
|  | affluence | poor | 4 | affluent | 2 |  |  |
| **Social support** |  | no body to talk with | 1 |  |  |  |  |
|  |  | few friends | 1 |  |  |  |  |
|  |  | no constant companions from children | 1 |  |  |  |  |
|  |  | bad marital relationship | 2 |  |  |  |  |
| **Personal traits** |  | closed | 7 | open | 1 |  |  |
|  |  | no hobbies | 6 | many hobbies | 3 |  |  |
|  |  | lazy | 3 | industrious | 6 |  |  |
|  |  |  |  |  |  | self-centered | 3 |
|  |  |  |  |  |  | do not care about the self | 14 |
|  |  | dependent | 4 | independent | 3 |  |  |
|  |  | dirty | 1 | clean | 1 |  |  |
|  |  | nagging | 1 |  |  |  |  |
|  |  |  |  | brave | 1 |  |  |
|  |  |  |  |  |  | thrift | 7 |

**Supplementary Table 3. Comparison of reasons for unhappy and happy late-life (*N* = 45).**

|  | **Reasons for unhappy late-life** | ***N*** | **Reasons for happy late-life** | ***N*** |
| --- | --- | --- | --- | --- |
| **Physical/mental health** | bad body health | 6 | good body health | 11 |
|  | feel lonely | 1 | feel of belonging | 3 |
|  | feel losing control | 1 | being respected | 2 |
|  | **Total** | **8** |  | **13** |
| **QoL** | bad economy | 6 | good economy and welfare | 5 |
|  | hard work | 3 | do not need to work | 5 |
|  | nothing to do | 1 | rich life | 4 |
|  | life stress events | 4 | quiet and peaceful | 1 |
|  |  |  | regular | 2 |
|  | **Total** | **11** |  | **15** |
| **Social support** | children unfilial | 25 | children filial | 21 |
|  | family conflicts | 8 | good family relationship | 7 |
|  | living alone | 4 | have companion | 1 |
|  |  |  | good neighborhood relationship | 3 |
|  |  |  | have friends | 7 |
|  | partner died | 1 |  |  |
|  | living in nursing home | 1 |  |  |
|  | **Total** | **31** |  | **27** |
| **Personal traits** | no hobbies | 3 | have hobbies | 15 |
|  | closed | 3 | keep pace with time | 2 |
|  | not content | 1 | content | 5 |
|  | pessimistic | 1 | optimistic | 5 |
|  | bad character | 3 | good character | 1 |
|  |  |  | have ability | 5 |
|  |  |  | have faith | 1 |
|  |  |  | be resilient | 1 |
|  | **Total** | **10** |  | **27** |
| **Society** | unfairness | 1 |  |  |
| **No elderly was unhappy** | |  |  | 4 |
| **Elderly were the same, not happier, nor unhappier** | | | | 1 |

**Supplementary Table 4. Comparison of living with to without children (*N* = 45).**

|  | **Better to live together** | ***N*** | **Worse to live together** | ***N*** | **Same** | ***N*** |
| --- | --- | --- | --- | --- | --- | --- |
| **Mental health** | happier | 12 |  |  | depends on children | 2 |
|  | having children to depend | 10 | unhappier | 1 | both lonely | 8 |
|  | less lonely | 6 |  |  |  |  |
|  | less sense of loss | 2 |  |  |  |  |
|  | less suffer from missing children | 1 |  |  |  |  |
|  | less need to worry about children | 1 |  |  |  |  |
|  | feel spiritually supported by children | 10 |  |  |  |  |
|  | feel safe | 1 |  |  |  |  |
|  | a feeling of being useful | 2 |  |  |  |  |
|  | a feeling of being superiority | 1 |  |  |  |  |
| **QoL** | colorful | 3 | being disturbed | 3 |  |  |
|  | things to do | 3 | add burden to elders | 3 |  |  |
|  | richer | 1 |  |  |  |  |
| **Social support** | good for family relationship | 2 | mother and daughter-in-law conflicts | 1 |  |  |
| **Personal traits** | more modern | 1 |  |  | depends on whether old adults can have theirs own lives | 1 |
| **Total** |  | **21** |  | **7** |  | **11** |

**Supplementary Table 5. Comparison of attitudes and coping strategies when parents/themselves are old (*N* = 45).**

|  | **When parents are old** | | ***N*** | **When themselves are old** | | ***N*** |
| --- | --- | --- | --- | --- | --- | --- |
| **Attitudes** | unacceptable | won't leave parents living alone | 16 | unacceptable | cannot be apart from children | 1 |
|  |  | worried about parents if left them alone | 1 |  |  |  |
|  |  | **Total** | **23** |  | **Total** | **1** |
|  | acceptable | it's evitable | 5 | acceptable | it's evitable | 4 |
|  |  | do not want to live with parents | 2 |  | do not want to live with children | 9 |
|  |  | parents have their own lives | 1 |  | do not need to live with children | 13 |
|  |  | parents now living without children nearby | 2 |  |  |  |
|  |  | parents could adapt to living without children | 2 |  | for the good of children | 9 |
|  |  | **Total** | **10** |  | **Total** | **34** |
|  | struggling |  | 3 | accept conditionally | as long as... | 10 |
|  |  |  |  | accept reluctantly | no other ways | 3 |
| **Coping** | “try my best” to balance needs of two sides | | 31 |  |  |  |
|  |  | visiting parents often | 20 |  |  |  |
|  |  | staying close to home | 6 |  |  |  |
|  |  | living in the same city | 3 |  |  |  |
|  |  | traveling with parents | 3 |  |  |  |
|  |  | calling parents on the phone | 3 |  |  |  |
|  |  | video calls with parents | 3 |  |  |  |
|  |  | asking others to take care of parents | 3 |  |  |  |
|  |  | buying things for parents | 1 |  |  |  |
|  | change parents | | 12 | educate children to be filial | | 2 |
|  |  | encouraging parents to have own lives | 9 |  | |  |
|  |  | persuade parents to move with children | 3 |  | |  |
|  | if-then strategy | | 10 |  |  |  |
|  | change themselves to live with parents | | 3 |  |  |  |
|  | will not live with parents | | 1 |  |  |  |
|  | reach an agreement with parents | | 1 |  |  |  |
|  |  |  |  | depends on themselves | | 29 |
|  |  |  |  |  | get hobbies | 22 |
|  |  |  |  |  | make friends | 10 |
|  |  |  |  |  | have material guarantee | 5 |
